# Diagnosis of paediatric TB using Xpert^®^ MTB/RIF Ultra on fresh respiratory samples

**DOI:** 10.5588/ijtld.22.0007

**Published:** 2022-09-01

**Authors:** I. Sabi, W. Olomi, E. Nkereuwem, T. Togun, M. P. Gomez, M. Sylla, B. Diarra, M. Sanogo, E. Sichone, H. Mahiga, F. Njeleka, A. O. Ebonyi, U. Egere, N. E. Ntinginya, M. Hoelscher, N. Heinrich, B. Kampmann, The Reach4KidsAfrica (R4KA) Consortium

**Affiliations:** 1 National Institute for Medical Research, Mbeya Medical Research Center, Mbeya, Tanzania; 2 Center for International Health, University Hospital, Ludwig Maximilian University (LMU) Munich, Germany; 3 Vaccines and Immunity Theme, Medical Research Council Unit The Gambia at the London School of Hygiene & Tropical Medicine, Banjul, The Gambia; 4 Department of Clinical Research, Faculty of Infectious and Tropical Diseases, London School of Hygiene & Tropical Medicine, London, UK; 5 Paediatrics Department, University Teaching Hospital Gabriel Toure, Bamako, Mali; 6 University Clinical Research Centre, University of Sciences, Techniques and Technologies of Bamako, Bamako, Mali; 7 Department of Paediatrics, Jos University Teaching Hospital, Jos, Nigeria; 8 Department of International Public Health, Liverpool School of Tropical Medicine, Liverpool, UK; 9 German Centre for Infection Research (DZIF), Partner Site Munich, Munich, Germany; 10 Division of Infectious Diseases and Tropical Medicine, University Hospital, LMU Munich, Germany

**Keywords:** *childhood* tuberculosis, pulmonary tuberculosis, diagnostics evaluation, Xpert MTB, RIF Ultra

## Abstract

**OBJECTIVE:**

To evaluate the diagnostic accuracy of Xpert^®^ MTB/RIF Ultra (Ultra) on fresh respiratory samples for the diagnosis of pulmonary TB (PTB) in children.

**METHODS:**

Between July 2017 and December 2019, children with presumed TB were prospectively enrolled at clinical sites in three African countries. Children were assessed using history, physical examination and chest X-ray. Sputum or gastric aspirate samples were analysed using Ultra and culture. The diagnostic accuracy of Ultra was calculated against culture as the reference standard.

**RESULTS:**

In total, 547children were included. The median age was 4.7 years, 77 (14.1%) were HIV infected and 77 (14.1%) had bacteriologically confirmed TB. Ultra detected an additional 20 cases in the group of children with negative culture results. The sensitivity of Ultra was 66.3% (95% CI 47–82), and the specificity was 95.4% (95% CI 89–99) when assessed against culture as the reference standard.

**CONCLUSION:**

Despite the improved performance of Ultra as compared to Xpert as was previously reported, its sensitivity remains sub-optimal for the detection of TB in children. Ultra detected additional 20 cases which otherwise could not have been detected by culture alone, suggesting that the latter is an imperfect reference standard.

## Abstract (fr)

**OBJECTIF:**

Évaluer la précision diagnostique de Xpert^®^ MTB/RIF Ultra (Ultra) sur des échantillons respiratoires frais pour le diagnostic de la TB pulmonaire (PTB) chez les enfants.

**MÉTHODES:**

Entre juillet 2017 et décembre 2019, des enfants présentant une TB présumée ont été inscrits de manière prospective dans des sites cliniques de trois pays africains. Les enfants ont été évalués à l’aide d’antécédents, d’un examen physique et d’une radiographie pulmonaire. Les échantillons d’expectoration ou d’aspiration gastrique ont été analysés à l’aide de l’Ultra et de la culture. La précision diagnostique de l’Ultra a été calculée par rapport à la culture comme norme de référence.

**RÉSULTATS:**

Au total, 547 enfants ont été inclus. L’âge médian était de 4,7 ans, 77 (14,1%) étaient infectés par le VIH et 77 (14,1%) avaient une TB confirmée bactériologiquement. L’Ultra a détecté 20 cas supplémentaires dans le groupe d’enfants dont les résultats de culture étaient négatifs. La sensibilité de l’Ultra était de 66,3% (IC 95% 47–82) et la spécificité de 95,4% (IC 95% 89–99) par rapport à la culture comme norme de référence.

**CONCLUSION:**

Malgré l’amélioration des performances de l’Ultra par rapport à l’Xpert, comme cela a été précédemment rapporté, sa sensibilité reste sous-optimale pour la détection de la TB chez les enfants. Ultra a détecté 20 cas supplémentaires qui n’auraient pas pu être détectés par la culture seule, ce qui suggère que cette dernière est un standard de référence imparfait.

TB remains a significant cause of death in children. In 2020, of the estimated 1.3 million TB deaths, 208,000 (16%) was estimated to occur among children aged 0–14 years.^1^ Childhood TB is primarily paucibacillary and presents with non-specific clinical and radiological findings. Sampling methods to obtain respiratory secretions in children, such as sputum induction (SI) and gastric aspirate (GA), are not straightforward to perform, and have suboptimal yield when used with polymerase chain reaction (PCR) or culture methods. As a result, delayed or missed diagnosis is still common in children.

Since 2013, the WHO has recommended the use of Xpert^®^ MTB/RIF (Xpert) assay (Cepheid, Sunnyvale, CA, USA) as the initial test for simultaneous detection of TB and rifampicin (RIF) resistance in children.^2^ This was supported by early evaluation studies which demonstrated improved accuracy for TB detection compared to smear microscopy in children.^3^,^4^ Despite the WHO recommendation, several studies have demonstrated sub-optimal sensitivity of Xpert for the diagnosis of TB in children.^5^–^9^ In previous systematic reviews and meta-analysis, the pooled sensitivity of Xpert in children was 62%^10^ as compared to 89%^11^ in adults.

The Xpert^®^ MTB/RIF Ultra (Ultra) assay (Cepheid) is an advanced version of the Xpert assay with improved sensitivity for simultaneous detection of TB and RIF resistance.^12^ The initial evaluation in adults demonstrated higher sensitivity of Ultra than Xpert.^13^ Available data from limited studies in children also indicated a higher sensitivity for Ultra.^14^–^16^ In a recent meta-analysis of Xpert and Ultra assays for active TB and RIF resistance in children, Ultra demonstrated a pooled sensitivity of 72.5% and specificity of 97.5%.^17^ Ultra was endorsed by the WHO in 2017 for the detection of TB disease and RIF resistance, and the same recommendations that apply for Xpert, has since been adopted by the guidelines.^18^ Recently, the WHO has further recommended the use of Ultra in GA and stool samples for the diagnosis of TB in children.^19^ However, the evidence base to support this practice in children remains small; only a few studies using archived frozen samples and limited by small sample size have been published to date.^14^–^16^ Data from large prospective studies using freshly collected samples are still lacking. We aimed to evaluate the diagnostic accuracy of Ultra using fresh respiratory samples obtained from consecutively recruited children in a diverse African setting.

## METHODOLOGY

### Study participants, setting, study design

This study was conducted as part of the Reach4Kids Africa Consortium at study sites in three sub-Saharan Africa countries: the National Institute for Medical Research in Mbeya, Tanzania; the Medical Research Council Unit The Gambia at the London School of Hygiene & Tropical Medicine, Fajara, The Gambia; and University Teaching Hospital Gabriel Toure, Bamako, Mali. Between July 2017 and December 2019, children aged <15 years with presumed TB were consecutively enrolled and followed up at outpatient childhood TB clinics at the respective institutions, as previously described.^20^ Children were eligible for enrolment if they had symptoms suggestive of pulmonary TB (PTB) characterised by persistent or unremitting cough for >2 weeks and any of weight loss/failure to thrive or persistent unexplained fever. Through clinical assessment and microbiological evaluations, children were categorised as having ‘Confirmed TB’, ‘Unconfirmed TB’ or ‘Unlikely TB’ by experienced clinicians based on the National Institutes of Health consensus statement.^21^

### Procedures

At enrolment, medical history and demographic information were obtained for each child. Anthropo-metric measurements, which included weight-for-age, length/height-for-age, and body mass index-for-age *Z*-scores were calculated using WHO 2007 reference standards.^22^ Clinical investigation included chest radiography and HIV testing in children whose HIV status was not known following each country‘s HIV testing algorithms. Other investigations to exclude TB were performed as per local hospitals guidelines.

Sputum or GA samples were collected from all children in accordance with the standard operating procedures of the site in question. In Tanzania, we collected only one sample type, i.e., sputum and two samples were obtained per child using SI with nebulised hypertonic saline. In The Gambia, one sample was obtained using SI and when not feasible, a GA sample was collected, while in Mali one sample was obtained by GA. At all sites, sputum samples were obtained by spontaneous expectoration in older children who were able to expectorate.

All available samples were processed using standardised protocols at each study site. Ultra assay was performed in accordance with the manufacturer’s recommendations on fresh samples. All available samples were also processed for liquid culture using Mycobacterial Growth Indicator Tube (Becton Dickinson, Franklin Lakes, NJ, USA) and solid culture on Löwenstein-Jensen medium. The presence of *Mycobacterium tuberculosis* in positive cultures was confirmed using Ziehl-Neelsen acid-fast staining and MPT64 antigen detection test (Abbott, Palatine, IL, USA) or MTBDR*plus* line-probe assays (Hain Lifesciences, Nehren, Germany).

### Statistical analysis

All consecutively enrolled children were included in the primary and sub-group analyses. Data were double-entered into a MS Access database (Microsoft Redmond, WA, USA), compared and corrected for data entry errors.

To characterise the study population, data were summarised by frequency and proportions for categorical variables and medians and interquartile ranges for continuous variables. We calculated diagnostic accuracy (sensitivity, specificity) of Ultra against culture as a microbiological reference standard (MRS). We also calculated the sensitivity of Ultra against an extended microbiological reference standard (eMRS), defined as positive results of both Ultra and/or culture, in a sub population of children with confirmed TB, as was recently suggested.^23^ As positive Ultra results led to the initiation of TB treatment, children with corresponding negative culture results could not be classified as Unlikely TB disease; information on specificity could therefore not be presented due to inclusion bias.

For sub-group analysis, we stratified participants by country, smear results, HIV status, age and nutritional status. We estimated the pooled sensitivity, specificity and 95% confidence intervals (CIs) using the Bayesian bivariate random-effects meta-analysis to account for the possible effects of heterogeneity across the subgroups. All data were analysed using R statistical software v4.1.1 (R Computing, Vienna, Austria).

### Ethical approval and informed consent

The study was approved by the Tanzanian Medical Research Coordinating Committee, Dar-es-Salaam, Tanzania; the Gambian Government and MRC Joint Ethics committee, Banjul, the Gambia; and Ethics committee of the Faculty of Medicine, Pharmacy and Dentistry of the University of Sciences, Techniques and Technologies of Bamako, Bamako, Mali. Written informed consent was obtained from a literate parent or legal guardian. In the case of illiteracy, informed oral consent was attested by an independent witness. Older children additionally provided assent for study participation.

## RESULTS

A total of 671 children with presumed TB were recruited; 134 children were subsequently excluded from the analysis, including 14 with unknown TB status, 37 with no valid Ultra and/or culture results, and 83 in whom culture had not been performed. Ultra and culture results were obtained in 547 children comprising 189 from Tanzania, 116 from Mali and 242 from The Gambia. The study flow diagram shows the flow of participants according to TB test results and case definition categories (Figure).

**Figure:**
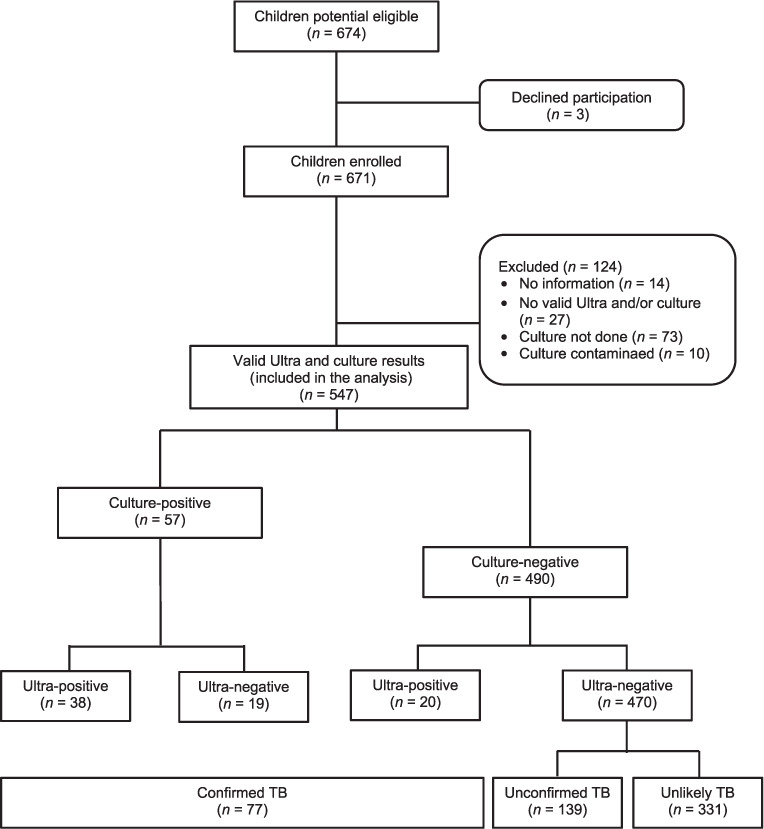
Study flow diagram by TB case definition. Children were categorised using the revised classification of intrathoracic TB for evaluation of studies in children as follows: ‘Confirmed TB group’ defined as a sub-group of children who tested positive for TB on culture, Ultra or both; ‘Unconfirmed TB group’ defined as a sub-group of children with no bacteriological confirmation but had suggestive signs and symptoms or suggestive radiological findings; and ‘Unlikely TB group’ defined as a sub-group of children with no bacteriological confirmation and the criteria for unconfirmed TB not met.

The median age was 4.7 years and 307 (56.1%) were males. Overall, 77/547 (14.1%) of the enrolled children were HIV-infected and 112 (20.9%) were severely malnourished. Children enrolled from Mali had the highest prevalence for both HIV (26.7%) and severe malnutrition (36%). Among all children, 460 (84.3%) had TB tests performed on sputum samples, while 86 (15.5%) TB tests were performed on GA samples. Overall, 77 (14.1%) had microbiologically Confirmed TB, 139 (25.4%) Unconfirmed TB and 331 (60.5%) Unlikely TB. There were no positive results for drug resistance either on Ultra or culture. Detailed demographic and clinical characteristics are presented in Table 1.

**Table 1:** Baseline characteristics of study participants

Characteristics	Tanzania (*n* = 189) *n* (%)	Mali (*n* = 116) *n* (%)	Gambia (*n* = 242) *n* (%)	Total (*n* = 547) *n* (%)
Age, years, median [IQR]	3.4 [1.5–7.2]	5 [2.2–10]	5.6 [2.3–8.5]	4.7 [2.0–8.5]
Age, years				
0–1	31 (16.4)	12 (10.3)	20 (8.3)	63 (11.5)
1–5	92 (48.7)	39 (33.6)	93 (38.4)	224 (41.0)
5–10	40 (21.2)	34 (29.3)	90 (37.2)	164 (30.0)
10–14	26 (13.8)	31 (26.7)	39 (16.1)	96 (17.5)
Male sex	103 (54.5)	72 (62.1)	132 (54.6)	307 (56.1)
HIV status				
Positive	21 (11.1)	31 (26.7)	25 (10.3)	77 (14.1)
Negative	163 (86.2)	85 (73.3)	211 (87.2)	459 (83.9)
Unknown	5 (2.7)	0	6 (2.5)	11 (2.0%)
BCG scar				
Yes	170 (90.0)	0	193 (79.8)	363 (66.4)
No	19 (10)	0	44 (18.2)	63 (11.5)
No information	0	116 (100)	5 (2.1)	121 (22.1)
Malnutrition status				
Severe	32 (16.9)	40 (36.0)	40 (16.9)	112 (20.9)
Moderate	24 (12.7)	24 (21.6)	57 (24.1)	105 (19.6)
Mild	87 (46.0)	41 (36.9)	121 (51.1)	249 (46.4)
Normal	46 (24.3)	6 (5.4)	19 (8.0)	71 (13.2)
Previously treated TB				
Yes	7 (3.7)	4 (3.5)	5 (2.1)	16 (2.9)
No	182 (96.3)	112 (96.5)	237 (97.9)	531 (97.1)
Type of specimen				
Spontaneous	48 (25.5)	31 (26.7)	36 (14.9)	115 (21.0)
Induced	140 (74.5)	0	201 (83.1)	341 (62.3)
Gastric aspirate	0	81 (69.8)	5 (2.1)	86 (15.8)
Culture-positive	21 (11.1)	23 (19.8)	13 (5.4)	57 (10.4)
Xpert Ultra-positive	19 (10.1)	23 (19.8)	16 (6.6)	58 (10.6)
Diagnostic classification				
Confirmed TB	25 (13.2)	31 (26.7)	21 (8.7)	77 (14.1)
Unconfirmed TB	23 (12.2)	69 (59.5)	47 (19.4)	139 (25.4)
Unlikely TB	141 (74.6)	16 (13.8)	174 (71.9)	331 (60.5)

IQR = interquartile range; BCG = bacille Calmette-Guérin.

Among 547 children, Ultra was positive in 58 children (10.6%), and culture was positive in 57 (10.4%) children. Among children with positive Ultra results, 38 were in the culture-positive group, while 20 were in the culture-negative group (Figure). Of those with positive Ultra but negative culture results, 8 were HIV-positive and 2 were previously treated for TB. Among the two children who were previously treated for TB, one was a HIV-positive, 13-year-old from Tanzania who was treated for TB in the past 1 year, and the other was a HIV-positive 8-year-old from Mali who was previously treated for TB in the past 5 years.

Using culture as MSR, Ultra sensitivity was 66.3% (95% CI 47–82) and specificity was 95.4% (95% CI 89–99) (Table 3). Using eMRS, we calculated the Ultra sensitivity in a sub-group of children with positive culture and/or Ultra results combined. With this approach, Ultra sensitivity against eMRS was 76% (95% CI 61–88), while that of culture was 74% (95% CI 53–89) (Table 2).

**Table 2:** Sensitivity of Ultra using extended microbiological reference standard

Characteristics	*n*-positive/*N*	Sensitivity % (95% CI)
Test		
Xpert Ultra	58/77	75 (60–87)
Culture	57/77	74 (53–89)
Country (Xpert Ultra)		
Gambia	16/21	76 (61–88)
Tanzania	19/25	75 (61–86)
Mali	23/31	74 (61–85)
Country (culture)		
Gambia	13/21	68 (47–83)
Tanzania	21/25	78 (63–91)
Mali	23/31	74 (60–86)

CI = confidence interval.

**Table 3:** Diagnostic accuracy of Ultra using culture as microbiological reference standard

Characteristics	Sensitivity % (95% CI)	*n*-positive/*N*	Specificity % (95% CI)	*n*-negative/*N*
Overall				
Ultra	66.3 (47.0–82.2)	38/57	95.4 (88.7–98.7)	470/490
Country				
Gambia	60.0 (12.0–95.2)	8/13	94.8 (72.0–99.7)	221/229
Mali	64 (14.5–95.7)	15/23	88.9 (49.7–99.2)	85/93
Tanzania	69.1 (19.0–96.8)	15/21	96.1 (79.3–99.8)	164/168
HIV status				
HIV-positive	57.1 (24.4–85.4)	4/7	88.4 (76.8–95.9)	62/70
HIV-negative	67.6 (51.9–80.8)	34/50	97.0 (93.9–98.9)	397/409
Age, years				
0–1	58.7 (20.6–89.8)	3/5	97.8 (90.7–99.8)	57/58
1–5	47.9 (26.3–69.9)	11/23	97.2 (92.6–99.3)	196/201
5–10	87.5 (58.4–98.7)	9/10	93.1 (84.3–98.0)	144/154
10–14	78.4 (56.2–92.9)	15/19	94.8 (86.3–98.9)	73/77
Malnutrition status				
Severe	76.0 (56.0–91.0)	17/22	93.0 (86.0–98.0)	84/90
Moderate	47.0 (21.0–75.0)	5/11	97.0 (92.0–99.0)	92/94
Mild	64.0 (42.0–83.0)	13/20	95.0 (90.0–98.0)	218/229
Normal	71.0 (26.0–97.0)	3/4	98.0 (92–100)	66/67
Type of specimen				
Spontaneous sputum	74.0 (54.0–88.0)	20/27	94.0 (87.0–98.0)	83/88
Induced	68.0 (42.0–88.0)	11/16	97.0 (94.0–99.0)	320/329
Gastric aspirate	48.0 (21.0–75.0)	7/14	90.0 (79.0–97.0)	66/72

CI = confidence interval.

Against MRS, Ultra sensitivity was higher in children enrolled in Tanzania (69.0%, 95% CI 19.0–97.0) than in those enrolled in The Gambia (60.0%, 95% CI 12.0–95.0) and Mali (64.0%, 95% CI 15.0–96.0). By HIV status, Ultra sensitivity was higher in HIV-negative children (76.6%, 95% CI 51.9–80.8) than in HIV-positive children (57.1%, CI 24.4–85.4). Ultra sensitivity was higher in the 5–10 (87.5%, 95% CI 58.4–98.7) and 10–14 (78.4%, 95% CI 56.2–92.9) year groups than in lower age groups. By nutrition status, Ultra sensitivity was higher in severely malnourished children (76.0%, 95% CI 56.0–91.0). Differences in sensitivities in the stratified analysis were not statistically significant. Against the MRS, Ultra specificity was lower in children enrolled in Mali (89.0%, 95% CI 50–99.0), in HIV-positive children (88.4%, 95% CI 76.8–95.9) and in children who produced GA samples (90.0%, 95% CI 79.0–97.0) (Table 3).

## DISCUSSION

We prospectively assessed the performance of Ultra using fresh respiratory samples in 547 children with presumed TB in three African countries. In our study, the sensitivity of Ultra was 66.3% in children with culture-confirmed TB, similar to earlier publications using stored samples where sensitivities of respectively 64.3%^14^ and 67.5%^15^ were observed. In a recent study involving hospitalised children,^16^ the sensitivity of Ultra was 74% when a single SI sample was used, which is higher than in our study. This study enrolled hospitalised children, therefore, possibly had far advanced TB diseases with higher likelihood of microbiological confirmation. In our study, Ultra detected TB in 20 additional children who were culture-negative. In these children, only two had a history of previously treated TB, which could have led to a suspicion of false-positive Ultra results. All children with Ultra-positive, culture-negative results had suggestive TB symptoms and improved on TB treatment, which suggests that they may well have had TB. In our study, the specificity of Ultra was 95.4%, lower than in the recent meta-analysis and systemic review,^17^ but similar to a recent study in Uganda.^24^

Using the eMRS, the sensitivity of Ultra was 75% and that of culture was 74%, suggesting that Ultra detects additional cases that would otherwise not be detected on culture and vice versa. These findings are similar to a very recent study in Uganda, which enrolled children with minimal PTB disease,^24^ in which sensitivities of 72% was reported for Ultra and 63.8% for MGIT culture, highlighting the need to combine Ultra and culture, where available, in order to improve TB case detection in children.

Studies have demonstrated that the sensitivity of Ultra improves with the number of samples tested. In earlier evaluation studies of Ultra in children, the sensitivity of 75%^14^ and 72%^15^ were demonstrated when more than one sample was used. Similarly, in a very recent study, the sensitivity of Ultra increased to 87.5% when two nasopharyngeal samples were combined with one SI.^16^ In our study, the majority of children had only one sample collected (only one sample per child was collected in Mali and The Gambia, and two in Tanzania). This may explain the slightly higher sensitivity of Ultra in Tanzania than the other two countries, although these differences were not statistically significant. We think our findings reflect the real-life scenario in many limited-resource countries, where often only one sputum sample is collected due to the high cost of the test, its labour-intensive nature and unpleasant sample collection methods in children. This further underlines the relevance of the recent WHO recommendation on the use of Ultra on stool samples to improve TB detection in children.^19^

In a recent meta-analysis and systemic review, the sensitivity of the Xpert assay was highest in GA samples, followed by sputum samples, and lowest in the nasopharyngeal (NPA) samples.^17^ Current evidence on the diagnostic accuracy of Ultra is predominantly from SI or NPA samples; data on Ultra sensitivity in GA samples are still lacking. In our study, we observed that Ultra sensitivity was lower in GA samples (48.0%) than in SI samples (68.0%), and highest in spontaneous sputum samples (74.0%). Spontaneous sputum samples were obtained in older children who were able to expectorate, which can explain the higher sensitivity in this group. Both SI and GA samples were obtained in younger children; the difference in sensitivities can be explained by the differences in the composition of the two groups, i.e., GA samples were collected predominantly in children from the Mali site with a large proportion of HIV-positive children, and overall, GA was only performed in a small number of children (15%) as compared to those who provided SI samples.

The strength of our study is its large sample size and large number of microbiologically confirmed TB cases. It is a prospective evaluation study of Ultra using fresh samples rather than previously frozen respiratory samples. The available evidence of Ultra diagnostic performance in children to date is mainly from South Africa and from East African countries.^14^–^16^,^24^ In our study, we extended the coverage to two West African countries known to have a high prevalence of *M. africanum*.^25^ We have demonstrated that Ultra performs equally well in different settings, as our findings are generally comparable to what have been previously reported.

Our study had some limitations. We were not able to do head-to-head comparisons of Ultra against Xpert due to the secondary nature of our study. All studies that have been conducted to date on Ultra have demonstrated that it performs better than Xpert for TB diagnosis in children, and the reported sensitivities and specificities of Ultra are relatively similar to our findings. Furthermore, as the semi-quantitative readout of the Ultra results was not reported, further analysis of trace call results, especially in 20 children with positive Ultra, but culture-negative results, could not be performed. All children with discordant results responded well to TB treatment, which supports the diagnosis of TB. In high TB burden settings, the current WHO recommendation supports the initiation of TB therapy based on trace call results in persons with HIV, children and those with extrapulmonary TB, as not treating them is a very high-risk approach with possible serious consequences.

Despite the improvement of diagnostic accuracy of Ultra as compared to Xpert as has been previously seen, Ultra sensitivity remains sub-optimal for the detection of TB in children. Ultra detected an additional 20 cases which would otherwise not have been detected using culture alone, suggesting that culture is an imperfect reference standard.
